# Supplementary material for: Breakdown of self-incompatibility due to genetic interaction between a specific S-allele and an unlinked modifier
Source: Nat Commun. 2023 Jun 9;14:3420. doi: 10.1038/s41467-023-38802-0 (PMC10256779; doi:10.1038/s41467-023-38802-0)
Supplement: Supplementary file 1 — Supplementary Information [file 41467_2023_38802_MOESM1_ESM.pdf]

Breakdown of self-incompatibility due to genetic interaction between a specific *S*-allele and an unlinked modifier

Li *et al*.

Supplementary Table 1. Between cross-type comparisons of mean SC-index. Contrasts were specified using a contrast matrix and significances evaluated using post-hoc two-sided *z*-tests.

**Contrasts**	**Estimate**	***z***	***P***
C1: BP$_{♀SI \times ♂SI}$ *vs* WP$_{♀SI \times ♂SI}$	-0.04	-1.63	0.53
C2: BP$_{♀SC \times ♂SC}$ *vs* WP$_{♀SC \times ♂SC}$	0.05	4.05	<0.001
C3: BP$_{♀SI \times SI}$ *vs* BP$_{♀SC \times ♂SC}$	-0.63	-19.68	<0.001
C4: BBS$_{♀SI \times ♂SC}$ *vs* BBS$_{♀SC \times ♂SI}$	-0.08	-2.08	0.26
C5 BBS *vs* BP	-0.02	-1.10	0.87
C6: BBS *vs* BP$_{♀SI \times ♂SI}$	0.33	14.90	<0.001
C7: BBS *vs* BP$_{♀SC \times ♂SC}$	-0.30	-14.37	<0.001
C8: BBS$_{♀SI \times ♂SC}$ *vs* BP			
C9: BBS$_{♀SC \times ♂SI}$ *vs* BP			
C10: BBS$_{♀SI \times ♂SC}$ *vs* BP$_{♀SI \times ♂SI}$		Tests are irrelevant [a]	
C11: BBS$_{♀SC \times ♂SI}$ *vs* BP$_{♀SI \times ♂SI}$			
C12: BBS$_{♀SI \times ♂SC}$ *vs* BP$_{♀SC \times ♂SC}$			
C13: BBS$_{♀SC \times ♂SI}$ *vs* BP$_{♀SC \times ♂SC}$			

Notes: Negative estimates indicate that the first cross type in the contrast had a lower mean SC-index value than the second one, and positive estimates indicate the opposite. Significant effects are highlighted in bold. WP: Within Population. BP: Between Population. BBS: Between Breeding System (by definition also between populations). Subscripts indicate the breeding system (SI: Self-Incompatible; SC: Self-Compatible) of the maternal (♀) and paternal (♂) cross-partners. Source data are provided as a Source Data file.

[a] Given that the SC-index of progeny did not depend on the maternal/paternal breeding system (i.e., C4 was not significant), contrasts C8-C13 are irrelevant and thus not reported. However, as they were part of the design and could not be omitted *a priori*, significance levels of individual contrasts have conservatively been corrected for all 13 contrasts.

Supplementary Table 2. Breeding system phenotype frequencies based on SC-index values for cross-progeny for each cross-type.

Cross-type	Breeding system phenotype				Totals
	Self-incompatible (SI)	Intermediate between SI and SC		Self-compatible (SC)	
	SC-index < 0.25	0.25 ≤ SC-index ≤ 0. 5	(0.5< SC-index ≤ 0.75)	SC-index > 0.75	
**Crosses within breeding system, within population (WP) and between population (BP)**					
WP♀SI × ♂SI	51	10	9	5	75
BP♀SI × ♂SI	168	21	14	7	210
WP♀SC × ♂SC	0	1	5	78	84
BP♀SC × ♂SC	1	0	7	222	230
**Crosses between breeding systems (BBS)**					
For crosses involving all six selfing populations					
BBS ♀SI × ♂SC	202	28	25	200	455
BBS♀SC × ♂SI	161	15	23	250	449
BBStotal	363	43	48	450	904
Subset of crosses involving SC populations fixed for S_1 (RON and PTP)					
BBS ♀SI×♂SC	96	14	10	42	162
BBS♀SC×♂SI	86	8	13	64	171
BBStotal	182	22	23	106	333
Subset of crosses involving SC populations fixed for S_{19} (TC, TSSA, LPT and KTT)					
BBS ♀SI×♂SC	83	7	13	147	250
BBS♀SC×♂SI	58	7	10	173	248
BBStotal	141	14	23	320	498

Notes: Plants with an intermediate SC-index (0.25 ≤ SC-index ≤ 0.75) could not be phenotyped unambiguously.

Supplementary Table 3. Background information for the six selfing and six outcrossing *A. lyrata* populations from which we used seeds.

Population code	Location, lake, state/Province, country	Population coordinates		Predominant breeding system (proportion of SC individuals) [a]	Outcrossing rate T_m[a]
		Latitude	Longitude		
**IND**	Indiana Dunes National Lakeshore, Lake Michigan, Indiana, USA	N 41°37'17"	W 87°12'44"	SI (0.14)	0.99
**MAN**	Manitoulin Island, Georgian Bay, Ontario, Canada	N 47°39'54"	W 82°15'52"	SI (0.13)	0.83
**SBD**	Sleeping Bear Dunes National Lakeshore, Lake Michigan, Michigan, USA	N 44°56'20"	W 85°52'13"	SI (0.00)	0.94
**TSS**	Tobermory Singing Sands, BPNP, Lake Huron, Ontario, Canada	N 45°11'33"	W 81°35'02"	SI (0.00)	0.91
**PCR**	Port Crescent State Park, Lake Huron, Michigan, USA	N 44°00'15"	W 83°04'26"	SI (0.00)	0.98
**PIN**	Pinery Provincial Park, Lake Huron, Ontario, Canada	N 43°16'08"	W 81°49'53"	SI (0.00)	0.84
**PTP**	Point Pelee National Park, Lake Erie, Ontario, Canada	N 41°55'40"	W 82°30'51"	SC (1.00)	0.09
**RON**	Rondeau Provincial Park, Lake Erie, Ontario, Canada	N 42°15'41"	W 81°50'47"	SC (1.00)	0.28
**TC**	Tobermory Cliffs, Bruce Peninsula National Park, Georgian Bay, Ontario, Canada	N 45°14'30"	W 81°31'03"	SC (0.88)	0.18
**TSSA**	Tobermory Singing Sands Alvar, BPNP, Lake Huron, Ontario, Canada	N 45°11'27"	W 81°35'26"	SC (0.50)	0.41
**LPT**	Long Point Provincial Park, Lake Erie, Ontario, Canada	N 42°34'47"	W 80°23'15"	SC (1.00)	0.13
**KTT**	Kitty Todd State Nature Preserve, -, Ohio, USA	N 41°37'14"	W 83°47'15"	SC (1.00)	0.31

Notes: Seeds from natural populations were kindly provided by Barbara Mable allowing us to grow the parents of our crossing design (Fig. 1).

[a] Values from Foxe *et al.* [1].

Supplementary Table 4. Timeline and progeny numbers for the eight batches of cross-progeny.

Source	Batch number								Total
	1 (R1)	2 (R2)	3 (R3)	4 (R4)	5 (R5)	6 [a] (NK)	7 [b] (R6)	8 [a] (R7)	
**Time schedule**									
Sowing month/year	7/2015	10/2015	9/2016	2/2017	8/2017	8/2017	1/2018	2/2018	-
First pollination	9/2015	1/2016	11/2016	4/2017	10/2017	10/2017	4/2018	4/2018	-
Last pollination	3/2016	7/2016	4/2017	9/2017	4/2018	4/2018	8/2018	9/2018	-
**Seed and germination**									
Parental set	A, B, C	A, B, C	D, E, F	D, E, F	A, B, C	A, B, C, D, E, F	A, B, C	A, B, C	-
Number of families with at least one seed	446	446	452	452	430	211	269	204	2,437
Number of germinated seeds	419	427	204	206	326	155	164	138	2,039
**Self-Pollination**									
Number of plants that could be self-pollinated	276	196	202	206	320	145	146	112	1,603
Total number of self-pollinations (in principle, 10 self-pollinations per plant)	2,564	1,855	2,045	2,137	3,247	1,503	1,483	1,146	15,980
**Sterility testing**									
Number of tested plants	116	62	95	85	-	-	-	-	358
Fertile	114	55	87	75	-	-	-	-	331 (93.0%)
Completely sterile	0	4	3	3	-	-	-	-	10 (2.8%)
Only female sterile	0	0	5	7	-	-	-	-	12 (3.4%)
Only male sterile	2	3	0	0	-	-	-	-	5 (1.4%)

Notes: In each batch, we sowed one seed per seed-family. The work for seven batches (identified by R1 to R7) were mainly executed by Yan Li. The sixth batch (identified by NK) was mainly executed by Nadja Köhler.

[a] This batch only included progeny from between-breeding-system crosses.

[b] This batch included progeny from crosses between-breeding-system and within-population, but not between populations with the same breeding system.

Supplementary Table 5. Linear mixed model analysis to test the effect of maternal and paternal fruit length and their interaction on progeny fruit length after self-pollination in selfing populations.

Source		Progeny fruit length after selfing (Gaussian)			
**Fixed effects**	df	Estimate	SE	χ^2	*P*
Maternal fruit length after selfing (MFL)	1	0.44	0.34	7.49	0.006
Paternal fruit length after selfing (PFL)	1	0.50	0.34	11.99	<0.001
MFL:PFL	1	-0.01	0.01	0.28	0.60
**Random effects**			SD		
Maternal population	1		1.60		
Paternal population	1		1.33		
Maternal plant (nested in Maternal population)	1		1.08		
Paternal plant (nested in Paternal population)	1		0.94		
Batch code	1		1.09		
Residual	193		3.55		

Notes: We tested the significance of the fixed effects by comparing models with and without the tested term using likelihood-ratio tests. Significant effects are indicated in bold. We individually calculated fruit length of progeny plants and parental plants based on the median fruit length of multiple fruits per plant prior to analysis. Source data are provided as a Source Data file.

Supplementary Table 6. *S*-allele-specific primers for *SRK* gene sequencing.

As further confirmation of putative *S*-genotypes, we used *S*-allele-specific primers SRK_1, SRK_3, SRK_{13}, SRK_{19}, SRK_{20} and SRK_{39}. These specifically amplify alleles of the female self-recognition gene *SRK* (*S*-locus Receptor Kinase) with a specific reverse primer (in the case of S_1) or a general reverse primer (SLGR). Reaction mixtures included reagents obtained from Thermo Fisher Scientific (Carslsbad CA, USA): 2.5 mM $MgCl_2$, 10x PCR Buffer, 0.2 mM dNTP), 0.1 mM of primers, 0.5 units of Taq DNA polymerase and 0.5 µl of DNA template (approximately 50 ng/ml). The PCR was run on a TProfessional Basic Gradient thermocycler (Biometra, Jena, Germany) with a 3 min initial denaturation cycle at 94°C, 1 min at primer-specific annealing temperature ($T_{annealing}$), and 2 min extension at 72°C, followed by 34 cycles (30 s at 94°C, 30 s at $T_{annealing}$, 2 min at 72°C) and a 6 min final elongation at 72°C.

Target	Name	$T_{annealing}$	Sequence (5'-3')	Reference
**Forward (F) primers**				
SRK_1	qtALSRK01F	57 ºC	tcctacatcatcgcag	J. Bechsgaard, personal communication
SRK_3	13-3F2	50 ºC	gtataaactcgaaatgcctag	(Mable *et al.* [2])
SRK_{13}	13-13F1	57 ºC	tggagatgcaaaaatgggacg	(Mable *et al.* [2])
SRK_{19}	13-19F1	62 ºC	gagttttacctgagtttttcatc	(Mable *et al.* [2])
SRK_{20}	13-20F1*	57 ºC	aacaatgggaatacatggtctc	(Mable *et al.* [2])
SRK_{23}	13-23F1	57 ºC	aacaacagagactcgccaactc	(Mable *et al.* [2])
SRK_{39}	Ah18bmF1	57 ºC	cgagacgccgatcccagct	
**Reverse primers**				
General	SLGR	50-62 ºC	atcgacataaagatcttgacc	(Schierup *et al.* [3])
SRK_1	qtALSRK01R	57 ºC	attcaacggtgtcctc	J. Bechsgaard, personal communication

* This primer yielded a weak amplicon of the wrong length in samples with S_{20} based on B80 linkage.

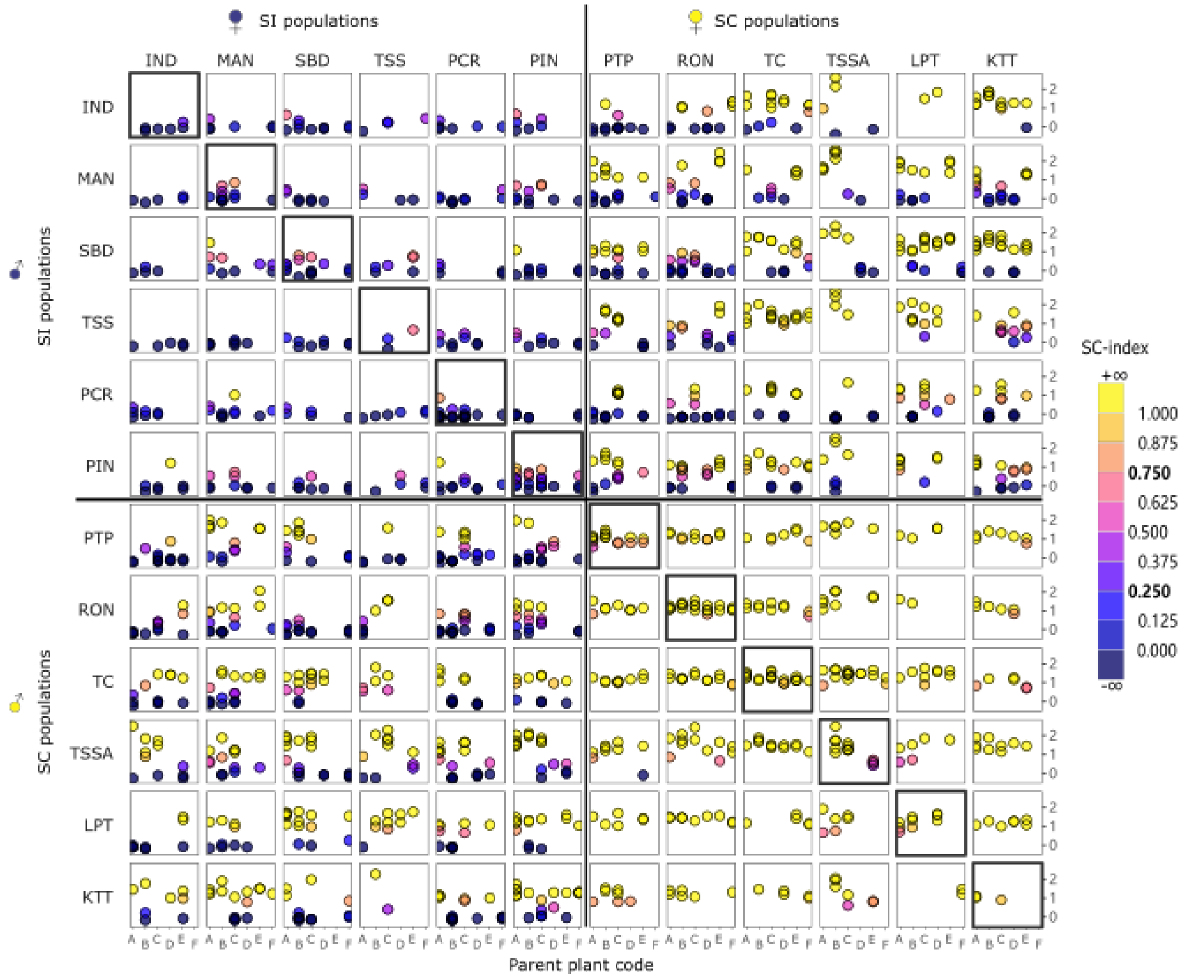

Supplementary Figure 1. SC-index values for individual cross-progeny by seed family. Crosses between populations were always done between parents with the same plant code (i.e. plants coded A were always crossed with another plant coded A, plants coded B with other plants coded B, et cetera, cf. crossing design in Fig. 1). Population coding corresponds to the coding in Supplementary Table 1. Diagonal cells marked with an extra outline represent progeny from within-population crosses, which resulted from crosses among plants coded A, B and C and among plants coded D, E and F. Source data are provided as a Source Data file.

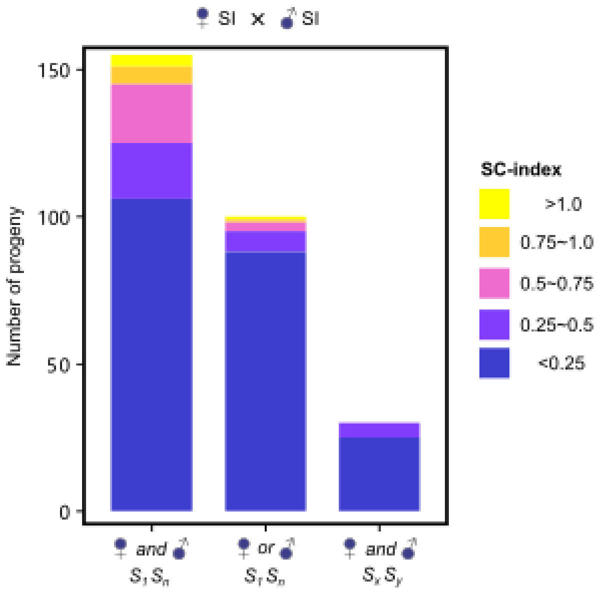

Supplementary Figure 2. Progeny SC-index for ♀SI × ♂SI crosses between plants from SI populations.

Presence or absence of S_1 in parents was inferred by PCR with primers specific for S_1 (Supplementary Table 5). Band presence after PCR would give the *S*-locus genotype S_1S_n, in which S_n represents any possible *S*-alleles (including S_1). Band absence after PCR would give S_xS_y, in which S_x and S_y represent *S*-alleles different from S_1. The first and second bar show progeny from crosses where both partners, respectively only one of the partners, had at least one copy of S_1. The third bar shows progeny from crosses where neither of the partners had S_1. Crosses were both within and between populations. Source data are provided as a Source Data file.

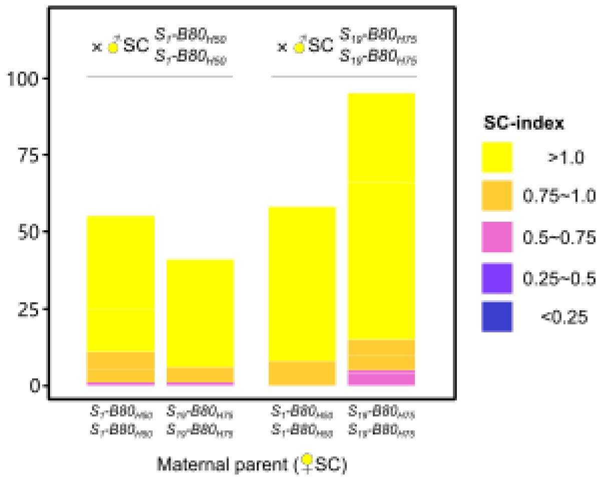

Supplementary Figure 3. Progeny SC-index for ♀SC × ♂SC crosses among plants from SC populations.

The crossed plants were homozygous for S_1-B80$_{H50}$ or S_{19}-B80$_{H75}$. Crosses were both within and between populations. Source data are provided as a Source Data file.

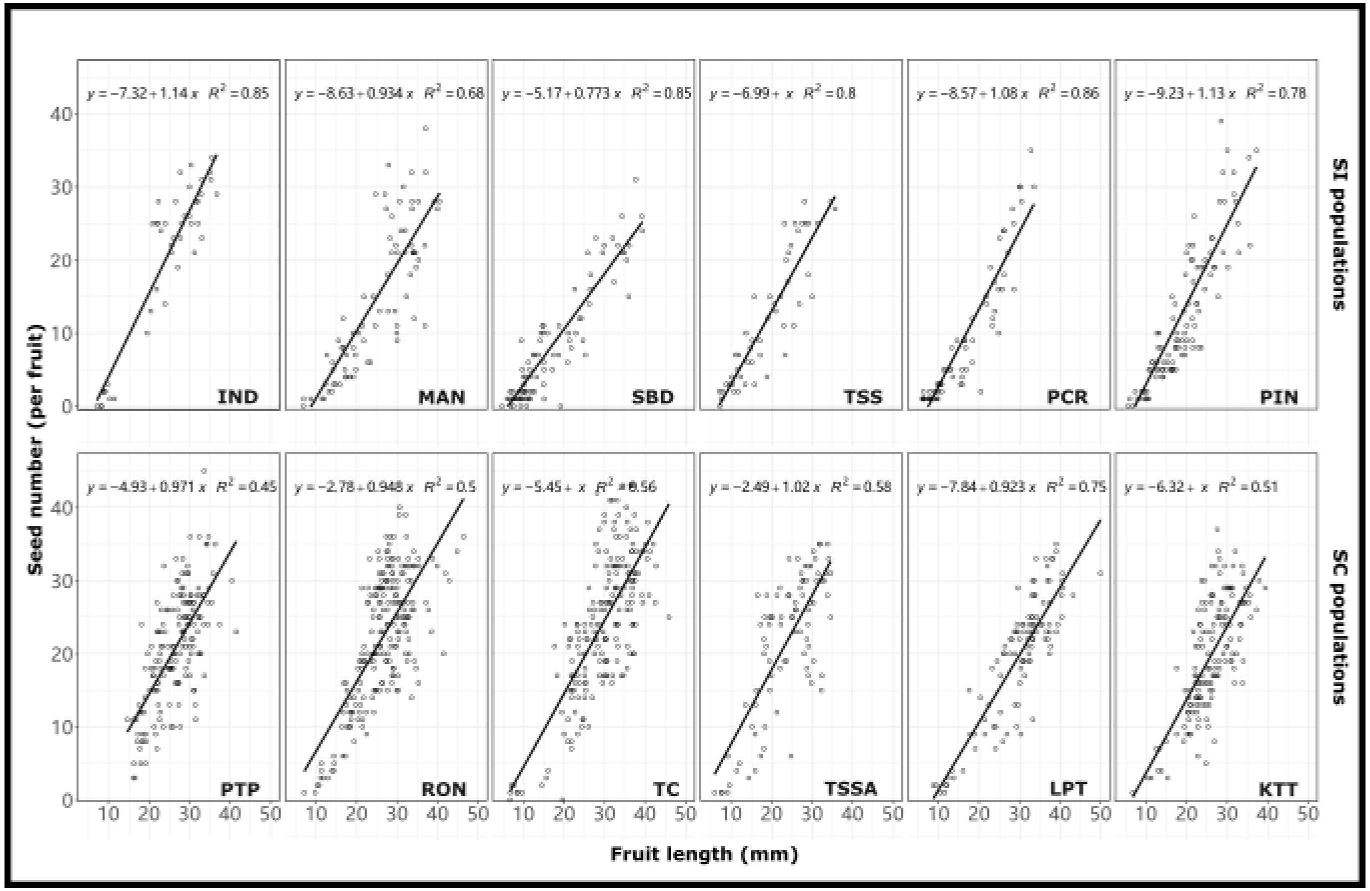

Supplementary Figure 4. Relationship between fruit length and seed number per fruit by maternal population.

For the subset of the fourth and fifth batches, we counted seeds per fruit for self-pollinated fruits, to test whether fruit length was a good predictor of seed set. Population codes correspond to those in Supplementary Table 1). Best-fit regression lines are shown along with their equation and proportion of explained variance R^2. Source data are provided as a Source Data file.

Supplementary references

1 Foxe, J. P. *et al.* Reconstructing origins of loss of self-incompatibility and selfing in North American *Arabidopsis lyrata:* A population genetic context. *Evolution* **64**, 3495-3510 (2010).

2 Mable, B. K., Schierup, M. H. & Charlesworth, D. Estimating the number, frequency, and dominance of *S*-alleles in a natural population of *Arabidopsis lyrata* (Brassicaceae) with sporophytic control of self-incompatibility. *Heredity* **90**, 422-431 (2003).

3 Schierup, M. H., Bechsgaard, J. S. & Christiansen, F. B. Selection at work in self-incompatible *Arabidopsis lyrata*. II. Spatial distribution of S haplotypes in Iceland. *Genetics* **180**, 1051-1059 (2008).
