# Supplementary material for: Breakdown of self-incompatibility due to genetic interaction between a specific S-allele and an unlinked modifier
Source: Nat Commun. 2023 Jun 9;14:3420. doi: 10.1038/s41467-023-38802-0 (PMC10256779; doi:10.1038/s41467-023-38802-0)
Supplement: Supplementary file 5 — Reporting Summary [file 41467_2023_38802_MOESM5_ESM.pdf]

nature portfolio

Corresponding author(s): Yan Li; Marc stift

Last updated by author(s): Apr 18, 2023

Reporting Summary

Nature Portfolio wishes to improve the reproducibility of the work that we publish. This form provides structure for consistency and transparency in reporting. For further information on Nature Portfolio policies, see our Editorial Policies and the Editorial Policy Checklist.

Statistics

For all statistical analyses, confirm that the following items are present in the figure legend, table legend, main text, or Methods section.

n/a	Confirmed	
☐	☒	The exact sample size (*n*) for each experimental group/condition, given as a discrete number and unit of measurement
☐	☒	A statement on whether measurements were taken from distinct samples or whether the same sample was measured repeatedly
☐	☒	The statistical test(s) used AND whether they are one- or two-sided<br>*Only common tests should be described solely by name; describe more complex techniques in the Methods section.*
☒	☐	A description of all covariates tested
☐	☒	A description of any assumptions or corrections, such as tests of normality and adjustment for multiple comparisons
☐	☒	A full description of the statistical parameters including central tendency (e.g. means) or other basic estimates (e.g. regression coefficient) AND variation (e.g. standard deviation) or associated estimates of uncertainty (e.g. confidence intervals)
☐	☒	For null hypothesis testing, the test statistic (e.g. *F*, *t*, *r*) with confidence intervals, effect sizes, degrees of freedom and *P* value noted<br>*Give P values as exact values whenever suitable.*
☒	☐	For Bayesian analysis, information on the choice of priors and Markov chain Monte Carlo settings
☐	☒	For hierarchical and complex designs, identification of the appropriate level for tests and full reporting of outcomes
☒	☐	Estimates of effect sizes (e.g. Cohen's *d*, Pearson's *r*), indicating how they were calculated

Our web collection on statistics for biologists contains articles on many of the points above.

Software and code

Policy information about availability of computer code

Data collection

No software was used for data collection.

Data analysis

All statistical analyses were performed using R version 4.2.3.

We used linear mixed effects models implemented in the lme function of the ‘nlme’ package (version number: 3.1.159)and we transformed the SC-index, which ranged from -0.39 to 2.71, by adding 1.39 to all values and subsequent natural log-transformation. To account for heterogeneity of variance, the model included a VarIdent variance structure.

We used the glht function in the multcomp package (version number: 1.4.20) to perform z-tests corrected for multiple comparisons.

We aligned the edited sequences to the B80-sequence library from Mable et al 2017 (https://datadryad.org/stash/dataset/doi:10.5061/dryad.832t8) using the Muscle algorithm (Edgar RC, 2004, MUSCLE: multiple sequence alignment with high accuracy and high throughput, Nucleic Acids Research 32: 1792-1797) as implemented in MEGA11 2004 (version 11).

For manuscripts utilizing custom algorithms or software that are central to the research but not yet described in published literature, software must be made available to editors and reviewers. We strongly encourage code deposition in a community repository (e.g. GitHub). See the Nature Portfolio guidelines for submitting code & software for further information.

Data

Policy information about availability of data

All manuscripts must include a data availability statement. This statement should provide the following information, where applicable:

- Accession codes, unique identifiers, or web links for publicly available datasets
- A description of any restrictions on data availability
- For clinical datasets or third party data, please ensure that the statement adheres to our policy

All data are available from Genbank and Figshare, as outlined in the data availability statement

Human research participants

Policy information about studies involving human research participants and Sex and Gender in Research.

Reporting on sex and gender	n/a
Population characteristics	n/a
Recruitment	n/a
Ethics oversight	n/a

Note that full information on the approval of the study protocol must also be provided in the manuscript.

Field-specific reporting

Please select the one below that is the best fit for your research. If you are not sure, read the appropriate sections before making your selection.

☐ Life sciences ☐ Behavioural & social sciences ☒ Ecological, evolutionary & environmental sciences

For a reference copy of the document with all sections, see nature.com/documents/nr-reporting-summary-flat.pdf

Ecological, evolutionary & environmental sciences study design

All studies must disclose on these points even when the disclosure is negative.

Study description

We did intra- and inter-population crosses using self-incompatible plants from six predominantly outcrossing populations and self-compatible plants from six predominantly selfing populations of North-American Arabidopsis lyrata. Selfing was previously known to be associated with specific S-locus genotypes, mainly (S1S1 and S19S19).

We determined 1,503 progeny breeding system by measuring 15,389 fruit lengths. We used linear mixed effects models to test for differences in SC-index between cross-types. With the SC-index as the dependent variable, the model fixed part included cross-type. To account for non-independence of cross-progeny derived from the same parents, the model random part included maternal population, maternal individual (nested in maternal population), paternal population, paternal individual (nested in parental population). Then, to infer S-locus genotypes, we targeted families that segregated for breeding system and for which parent S-locus genotypes could be inferred and genotyped 341 progeny to test dependence of the phenotype with S-locus genotype.

Based on these data, we conclude that the association between S1 and S19 and self-compatibility has a functional basis. For S1, the functional association is due to a genetic interaction with a modifier unlinked to the S-locus. The latter documents the first case of a breakdown of self-incompatibility that cannot be explained by loss-of-function mutations at the S-locus.

Research sample

We crossed 18 self-incompatible (SI) plants from six predominantly outcrossing populations (i.e. IND, MAN, PIN, PCR, SBD and TSS) and 18 self-compatible (SC) plants from six predominantly selfing populations (i.e. KTT, LPT, PTP, RON, TC and TSSA) of North-American Arabidopsis lyrata in all possible combinations. This design was replicated with another set of 18 SI and SC plants. The selfing populations include two populations (RON and PTP) with exclusively homozygotes for specificity S1, three populations (RON, TC and TSSA) with exclusively homozygotes for specificity S19 and one mixed population (TSSA) with S1S1, S19S19 and S27S27 homozygotes.

Sampling strategy

We recorded fruit length after self-pollination for F1 plants aiming for even representation of all available cross-combinations (seed families). However, because not all crosses resulted in seeds, not all sowing seeds germinated and not all germinated seeds survived to flowering stage, sample size per population-level cross-combination varied. On average, we obtained a replication of 11 per cross-combination (maximum: 21; minimum: 2; see Figure 3 for details), which was more than sufficient for our statistical inferences given the large underlying number of cross-combinations (see research sample for details on crossing design).

Data collection

All pollination work was done by transferring the pollen using a tweezer and rubbing it on the stigma. For collecting seeds, we collected ripe fruits by hand and stored them in small paper bags. For fruit length, we collected immature fruits, measured them with

a caliper and recorded values on score sheets. These data were mainly collected by Yan Li, Ekaterina Mamonova and Nadja Köhler.

To determine the S-locus genotype of progeny, we extracted DNA from silica-dried leaf material. We amplified the gene B80 by PCR and performed direct sequencing (outsourced to Eurofins Genomics, Konstanz, Germany). Obtained sequences were base-called and genotyped manually through alignment. These data were mainly collected by Ekaterina Mamonova and Nadja Köhler and Marc Stift.

Timing and spatial scale	Crosses to produce F1 progeny were conducted between March 2014 to December 2015.<br>Self-pollination trials to determine F1 progeny breeding system were conducted between July 2015 to September 2018.<br>B80 sequencing and S-locus genotyping of parents and progeny from 2019 to 2022.<br>All data was collected in the growth chambers and the molecular lab of the Ecology group at the university of Konstanz.
Data exclusions	We performed self-pollinations for a total of 1603 F1 progeny. Of those, 27 progeny was excluded due to putative male and/or female sterility. Of the remaining 1576 progeny, 55 were excluded because fewer than five self-pollinations could be performed, and a further 2 because fruit lengths had not been recorded. Of the remaining 1519 progeny, a further 16 was excluded after B80 genotyping due to suspected pollen-contaminations. Thus, a total of 100 out of 1603 progeny were excluded from data analysis.
Reproducibility	Reproducibility was ensured by the experimental design, which included replication at the level of mating system (six populations for each), at the level of population (multiple parents within each populations), at the family level (multiple F1 progeny per seed family) and at the individual level (at least five self-pollinations per F1 plant).
Randomization	For crossing, we randomly selected focal plants from each population, and randomly assigned them to their cross-partners.
Blinding	For F1 progeny, data collection was blind to sample identity for fruit length measurements, seed counts, and initial base-calling of B80 sequences.

Did the study involve field work? ☐ Yes ☒ No

Reporting for specific materials, systems and methods

We require information from authors about some types of materials, experimental systems and methods used in many studies. Here, indicate whether each material, system or method listed is relevant to your study. If you are not sure if a list item applies to your research, read the appropriate section before selecting a response.

Materials & experimental systems

n/a	Involved in the study	
☒	☐	Antibodies
☒	☐	Eukaryotic cell lines
☒	☐	Palaeontology and archaeology
☒	☐	Animals and other organisms
☒	☐	Clinical data
☒	☐	Dual use research of concern

Methods

n/a	Involved in the study	
☒	☐	ChIP-seq
☒	☐	Flow cytometry
☒	☐	MRI-based neuroimaging
